# Psychiatric comorbidities in pediatric epilepsy: a single-center retrospective study highlighting the burden of depressive symptoms

**DOI:** 10.3389/fneur.2026.1951989

**Published:** 2026-10-07

**Authors:** Arianna De Leonardis, Alberto Spalice, Alberto Verrotti, Isabella De Rosa, Simone Aloisio

**Affiliations:** 1 Pediatric Neurology Unit, Department of Maternal and Child Health and Urological Sciences, Policlinico Umberto I, Sapienza University of Rome, Rome, Italy; 2 Department of Pediatrics, University of Perugia, Perugia, Italy

**Keywords:** ADHD, depression, epilepsy, pediatrics, psychiatric comorbidity

## Abstract

**Introduction:**

Epilepsy in the pediatric population is frequently associated with psychiatric comorbidities. Depression is of particular clinical significance given its elevated incidence and substantial impact on quality of life, treatment adherence, and psychosocial functioning. The prevalence of depression in children with epilepsy ranges from 7% to 14%, considerably higher than the 1–5% reported in the general pediatric population. The relationship between epilepsy and depression appears bidirectional: epilepsy predisposes to depressive disorders, and depression may increase seizure risk, suggesting shared neurobiological pathways. This study aimed to assess the prevalence of psychiatric symptoms in pediatric patients with epilepsy, with particular focus on depressive symptomatology, and to examine associations with etiological and clinical variables.

**Methods:**

This retrospective study included 240 pediatric patients (aged 2–18 years) diagnosed with epilepsy between 2013 and 2024. Psychiatric symptoms were ascertained retrospectively from clinical records. Patients were categorized into two groups: those with an identified genetic and/or structural abnormality (G1, *n* = 60) and those without (G2, *n* = 180). Statistical analysis employed the chi-squared test and Student’s *t*-test (*p* < 0.05).

**Results:**

Psychiatric symptoms were identified in 30% of patients (72/240, 95% CI 24.2–35.8%). The most prevalent disorder was attention-deficit/hyperactivity disorder (ADHD; *n* = 30), followed by sleep disturbance/asthenia (*n* = 19), aggression/temper tantrums (*n* = 13), depressive/mood symptoms (*n* = 11), anxiety symptoms (*n* = 10), autism spectrum disorder (*n* = 6), eating behavior alterations (*n* = 4), self-harm (*n* = 4), adjustment/self-esteem features (*n* = 3), obsessive-compulsive disorder (OCD; *n* = 3), panic attacks (*n* = 2), and suicide attempts (*n* = 2). Psychiatric symptoms were significantly more prevalent in G1 (42%, 95% CI 29.2–54.1%) than in G2 (26%, 95% CI 19.7–32.5%; *p* = 0.03), corresponding to an odds ratio of 2.02 (95% CI 1.10–3.72). No significant associations were found between psychiatric symptoms and age, sex, family history, or treatment regimen.

**Discussion:**

Psychiatric comorbidities, including depressive symptomatology, are common in pediatric epilepsy and likely associated with underlying neurobiological factors. These findings support integrating neuropsychiatric screening, using validated instruments such as the Children’s Depression Inventory-2 (CDI-2) and the Neurological Disorders Depression Inventory for Epilepsy–Youth version (NDDI-E-Y), into routine epilepsy care.

## 1 Introduction

Epilepsy is among the most prevalent chronic neurological disorders in childhood, encompassing cognitive, behavioral, and psychiatric dimensions in addition to its core seizure manifestations. Worldwide, an estimated 10–15 million children under 15 years of age have active epilepsy, representing approximately 25% of the global epileptic population (1, 2). In Italy, the incidence of epilepsy is 48.35/100,000 new cases per year, with a peak in children under 15 years (50.14/100,000) and particularly in the first year of life (92.8/100,000) (3). Behavioral disorders are up to 4.8 times more common in children with epilepsy than in the general pediatric population, and approximately 2.5 times more common than in children with other chronic non-central nervous system diseases (4).

Among psychiatric comorbidities, depression is of particular concern because of its impact on psychosocial functioning, treatment compliance, and overall quality of life. The prevalence of depression in children with epilepsy ranges from 7 to 14%, considerably exceeding the 1–5% observed in the general pediatric population (5, 6). In pediatric patients, depressive symptoms frequently manifest atypically—presenting as irritability, aggression, psychomotor restlessness, somatic complaints, or academic decline rather than the classic low mood of adult depression (7, 8). The average duration of a depressive episode in childhood and adolescence is estimated at 7–9 months, with a recurrence rate of 40% after 2 years (9). Subclinical depressive symptoms in adolescence are strong predictors of major depressive disorder in adulthood (5).

The relationship between epilepsy and depression is complex and likely multifactorial. Neurobiological mechanisms, including alterations in limbic circuits, serotonergic dysfunction, and hypothalamic–pituitary–adrenal (HPA) axis dysregulation, are thought to contribute to both conditions (6, 10). Neuroimaging studies have demonstrated structural changes—particularly reductions in hippocampal and frontal lobe volumes—in both epilepsy and depression, suggesting overlapping pathological substrates (6, 10). Temporal lobe epilepsy (TLE) merits particular attention as the temporal lobe is closely linked to the limbic system, which regulates emotion, and epileptiform activity in this region may disrupt normal neurodevelopmental trajectories (11, 12). Approximately 44% of children with TLE are considered at risk for depression, and in 22% the depressive disorder meets criteria for a clinical diagnosis (13, 14). Furthermore, the co-occurrence of epilepsy and depression is associated with more pronounced hippocampal atrophy than either condition in isolation (6, 15). The high incidence of depressive symptoms even at the onset of epilepsy suggests that depression is not simply a psychological response to chronicity, but a condition inextricably linked to the biological mechanisms of seizure disorders (6). Despite increasing awareness, psychiatric comorbidities in pediatric epilepsy remain insufficiently investigated in clinical practice. The determinants of these comorbidities are not fully understood, and systematic screening is not yet standard of care. The present study tries to address this gap by reporting on a single-center retrospective cohort of 240 pediatric patients followed at a tertiary referral pediatric neurology unit in Italy over an 11-year period (2013–2024), a context in which large-scale Italian data on psychiatric comorbidities in pediatric epilepsy remain limited.

The objectives of this study were as follows:

- To evaluate the prevalence of psychiatric symptoms in pediatric epilepsy.
- To compare the prevalence of psychiatric symptoms between patients with epilepsy with an identified structural and/or genetic abnormality (G1) and those without an identified structural and/or genetic abnormality (G2).
- To assess the association between psychiatric symptoms and etiological as well as clinical variables.

## 2 Materials and methods

### 2.1 Study population and data collection

This retrospective study included pediatric patients diagnosed with epilepsy at the Pediatric Neurology Unit of Policlinico Umberto I from 2013 to 2024. The cohort comprised all consecutive patients seen at the unit during the study period who met the eligibility criteria detailed below; recruitment was not based on an *a priori* sample-size calculation.

Inclusion criteria were age between 2 and 18 years and a diagnosis of epilepsy, established in accordance with the operational clinical definition of the International League Against Epilepsy (ILAE) (16). Infants under 2 years of age were excluded, as assessment of psychiatric symptoms would not be feasible in this age group. A total of 240 patients met the inclusion criteria and, for analytical purposes, were categorized into two groups: those with an identified structural and/or genetic abnormality (G1, *n* = 60) and those without and identified structural and/or genetic abnormality (G2, *n* = 180). This bipartite grouping was adopted for descriptive and statistical comparison only and does not correspond to formal ILAE etiological classification, since structural and genetic etiologies may coexist in the same patient (17).

The following variables were collected: age at presentation, sex, family history of epilepsy, MRI findings, genetic testing results, antiseizure medication (ASM) therapy, and presence of psychiatric symptoms. The presence of psychiatric symptoms was determined retrospectively from documentation in the clinical record (e.g., problem list, clinical notes, referral letters); no validated screening instrument was systematically administered to this cohort during the study period. This ascertainment method is discussed further under Limitations. Patients with psychiatric symptoms (GPsy, *n* = 72) were compared with those presenting with isolated epilepsy (GE, *n* = 168) for all clinical and demographic variables.

### 2.2 Statistical analysis

The chi-squared test and Student’s *t*-test for independent samples were used, assuming unequal variances between groups. All analyses were performed using the Python programming language with the SciPy library. Statistical significance was set at *p* < 0.05. Prevalence estimates are reported with 95% confidence intervals (CIs) calculated using the normal approximation to the binomial distribution; the odds ratio (OR) for the G1–G2 comparison was calculated with its 95% CI using the Woolf logit method. For categories with small expected cell counts (e.g., suicide attempts, panic attacks, obsessive-compulsive disorder), results should be interpreted with caution given the reduced reliability of the chi-squared approximation; no correction for multiple comparisons was applied to the univariate analyses of clinical and demographic variable.

## 3 Results

### 3.1 Prevalence of psychiatric symptoms

The characteristics of the cohort are shown in Table 1.

**Table 1:** Baseline characteristics of the full study cohort (*n* = 240).

Characteristic	Total cohort (*n* = 240)
Age at visit, mean (years)	10.7
Age range (years)	2–18
Sex, male, *n* (%)	120 (50%)
Family history of epilepsy, *n* (%)	59 (25%)
G1—epilepsy with an identified genetical and/or structural abnormality, *n* (%)	60 (25%)
G2—epilepsy without an identified genetical and/or structural abnormality, *n* (%)	180 (75%)
Psychiatric symptoms present, *n* (%)	72 (30%)
ASM therapy—None, *n* (%)	42 (18%)
ASM therapy—Monotherapy, *n* (%)	160 (67%)
ASM therapy—Polypharmacy, *n* (%)*	38 (16%)

G1: epilepsy with an identified structural and/or genetic abnormality; G2: epilepsy without an identified structural and/or genetic abnormality (an analytical grouping used for the purposes of this study, rather than a formal ILAE etiological classification, as structural and genetic etiologies may coexist). ASM, antiseizure medication. Values marked * are derived from group-level data and rounded to the nearest integer. Exact sub-group counts were not separately tabulated during data extraction; these figures should therefore be considered approximate.

Psychiatric symptoms were present in 30% of patients (72/240, 95% CI 24.2–35.8%). Among these, depressive symptoms and mood disturbances constituted a clinically significant component, alongside ADHD, anxiety symptoms, and sleep disturbances. Cases of self-harm and suicide attempts, although limited in number, were also observed, highlighting the potential severity of mood-related symptoms. A detailed breakdown of the observed symptomatology revealed: ADHD (*n* = 30), sleep disturbance and asthenia (*n* = 19), aggression and temper tantrums (*n* = 13), depressive/ mood symptoms (*n* = 11), anxiety symptoms (*n* = 10), autism spectrum disorder (*n* = 6), eating behavior alterations including anorexia, bulimia, and hyperphagia (*n* = 4), self-harm (*n* = 4), adjustment disorders (including features of low self-esteem) (*n* = 3), obsessive-compulsive disorder (*n* = 3), panic attacks (*n* = 2), and suicide attempts (*n* = 2). This distribution reflects a wide spectrum of psychiatric manifestations, ranging from attentional and behavioral dysregulation to mood and affective symptoms. The full distribution of formal and symptoms is reported in Table 2.

**Table 2:** Distribution of psychiatric symptom categories among patients with epilepsy and psychiatric comorbidities (GPsy group, *n* = 72).

Category	*n*	% of GPsy group (*n* = 72)
Panel A: formal psychiatric diagnoses		
ADHD	30	41.7%
Autism spectrum disorder (ASD)	6	8.3%
Obsessive-compulsive disorder (OCD)	3	4.2%
Adjustment disorders	3	4.2%
Panel B: Psychiatric symptoms		
Sleep disturbance/asthenia	19	26.4%
Aggression/temper tantrums	13	18.1%
Depressive/mood symptoms	11	15.3%
Anxiety symptoms	10	13.9%
Eating behavior alterations	4	5.6%
Self-harm	4	5.6%
Panic attacks	2	2.8%
Suicide attempts	2	2.8%

Percentages refer to GPsy group total. Individual patients may have presented with more than one symptom category.

A significantly higher prevalence of psychiatric symptoms was found in G1 (42, 95% CI 29.2–54.1%) compared to G2 (26, 95% CI 19.7–32.5%, *p* = 0.03), corresponding to an odds ratio of 2.02 (95% CI 1.10–3.72). In absolute terms, 25 of 60 patients in G1 and 47 of 180 in G2 presented with psychiatric comorbidities. This statistically significant difference suggests that structural and/or genetic factors may confer additional vulnerability to psychiatric disorders, consistent with the hypothesis of a neurobiological basis for these comorbidities (Table 3).

**Table 3:** Prevalence of psychiatric symptoms in G1 (epilepsy with an identified structural and/or genetic abnormality) and G2 (epilepsy without an identified structural and/or genetic abnormality).

Variable	G1 (*n* = 60)	G2 (*n* = 180)	*p*-value
Psychiatric symptoms, *n*	25	47	
Psychiatric symptoms, %	42%	26%	**0.03**
No psychiatric symptoms, *n*	35	133	

Bold indicates statistical significance (*p* < 0.05).

The odds ratio for the association between G1 status and psychiatric symptoms was 2.02 (95% CI 1.10–3.72), corroborating the statistical significance reported above.

### 3.2 Clinical variables

No statistically significant associations were found between psychiatric symptoms and age, sex, family history of epilepsy, or treatment regimen.

Specifically: mean age at presentation was 10.55 years (SD = 4.30) in GE vs. 11.06 years (SD = 3.85) in GPsy (*p* = 0.37); males accounted for 49% in GE and 53% in GPsy (*p* = 0.67); family history of epilepsy was present in 27% of GE and 19% of GPsy (*p* = 0.25). Regarding ASM therapy, 16% of GE and 21% of GPsy were not receiving medication (*p* = 0.45); monotherapy was used in 70% of GE and 58% of GPsy (*p* = 0.10); polypharmacy in 14% of GE and 21% of GPsy (*p* = 0.25). None of these differences reached statistical significance (Table 4).

**Table 4:** Comparison of clinical and demographic variables between patients with isolated epilepsy (GE, *n* = 168) and patients with epilepsy and psychiatric symptoms (GPsy, *n* = 72).

Variable	GE-isolated epilepsy (*n* = 168)	GPsy-epilepsy + psychiatric symptoms (*n* = 72)	*p*-value
Age at visit, mean ± SD (years)	10.55 ± 4.30	11.06 ± 3.85	0.37
Age range (years)	2–18	4–18	—
Sex, male, *n* (%)*	82 (49%)	38 (53%)	0.67
Family history of epilepsy, *n* (%)*	45 (27%)	14 (19%)	0.25
ASM therapy			
None, (%)*	16%	21%	0.45
Monotherapy (%)*	70%	58%	0.10
Polypharmacy (%)*	14%	21%	0.25

Values marked * are derived from percentage × group size, rounded to the nearest integer. Exact sub-group counts were not separately tabulated during data extraction; these figures should therefore be considered approximate. ASM, antiseizure medication; SD, standard deviation. No statistically significant differences were observed across all variables.

## 4 Discussion

This study confirms a high prevalence of psychiatric comorbidities in pediatric patients with epilepsy, with a 30% rate consistent with the 21–60% range reported in the literature (18, 19). Dagar and Falcone (7) similarly emphasized the neurobiological underpinnings of psychiatric vulnerability in pediatric epilepsy, supporting the interpretation that the difference observed between G1 and G2 reflects underlying neuropathological susceptibility rather than epidemiological variation.

Notably, the prevalence of depressive/mood symptoms identified in this cohort (11/240, 4.6%, 95% CI 1.9–7.2%) is lower than the 7–14% range typically reported for pediatric epilepsy (5, 6), and the upper bound of our confidence interval only approaches the lower end of that range. This discrepancy likely reflects the retrospective, non-standardized ascertainment of psychiatric symptoms from clinical records rather than a true lower burden of depression, and reinforces the rationale for prospective screening with validated instruments such as the CDI-2 and NDDI-E-Y. This study’s observed prevalence of depressive symptoms was lower than reported elsewhere, most likely because of the non-standardized, retrospective way symptoms were ascertained here, rather than a genuinely lower burden in this population (see section 4.1). Nonetheless, depressive symptomatology was prioritized in this study for three reasons: it disproportionately contributes to serious adverse outcomes, including self-harm and suicide risk (20–25); it frequently presents atypically and is under-recognized in children (7, 8); and validated, epilepsy-specific screening instruments (CDI-2, NDDI-E-Y) exist for it but are not yet used routinely. For these reasons, depressive symptomatology is treated as a high clinical priority for systematic screening regardless of its numerical rank among this cohort’s comorbidities—ADHD was in fact the most prevalent comorbidity identified (*n* = 30, 41.7% of the GPsy group).

Although the present cohort included too few cases of self-harm (*n* = 4) and suicide attempts (*n* = 2) to test these associations directly within our own data, particular attention should be given to depressive symptomatology, which, even when subclinical, can significantly affect quality of life and increase the risk of adverse outcomes, including suicidal behavior. Notably, one in four children with epilepsy who screen positive for depression report suicidal symptoms (20), and the suicide rate in epilepsy patients is estimated to be up to five times higher than in the general population (21, 22). This elevated risk has been linked to serotonergic dysfunction and volumetric abnormalities in the orbital frontal gyrus and temporal lobe, brain regions involved in emotional regulation and impulse control (23, 24). Furthermore, for each additional psychiatric comorbidity, the odds of developing suicidal ideation increase by approximately 80% (25), underscoring the importance of comprehensive psychiatric assessment.

It should be noted that the present cohort included only 4 patients with self-harm and 2 with suicide attempts; these counts are too small to test the above associations within our own data, and the cited estimates of suicide risk are presented as context from the external literature rather than as findings of this study.

The higher prevalence of psychiatric symptoms in patients with genetic and/or structural abnormalities (G1) is consistent with current evidence implicating specific brain alterations—particularly in the temporal lobe, limbic system, and hippocampus—as predisposing factors for mood and anxiety disorders (6, 10). HPA axis hyperactivation and alterations in glutamate/GABA balance, shared pathological mechanisms between epilepsy and depression (26), may be particularly relevant in patients with structural or genetic etiologies. The co-occurrence of hippocampal atrophy observed in both conditions (6, 15) may further perpetuate depressive symptomatology in this subgroup.

The absence of significant associations with demographic and treatment variables examined is consistent with, though does not establish, a limited role for these factors relative to underlying neurobiological processes; given the cross-sectional, univariate nature of these comparisons and the possibility of unmeasured confounding, causal attribution cannot be inferred from these data. Nonetheless, ASM choice warrants careful consideration: drugs with more favorable neuropsychiatric profiles, such as lamotrigine and lacosamide, should be preferred over phenobarbital or topiramate when clinically appropriate (27, 28). From a clinical standpoint, prior literature recommends the implementation of validated screening instruments: the CDI-2 Children’s Depression Inventory-2 patients aged 7–17 years (29, 30) and the NDDI-E-Y (Neurological Disorders Depression Inventory for Epilepsy—Youth version) for those aged 12–17 years (31, 32). This approach is in keeping with the consensus-based recommendations of ILAE Psychiatric Pediatric Issues Task Force for the diagnosis and treatment of anxiety and depression in children and adolescents with epilepsy, which likewise endorse routine, validated screening as a core component of comprehensive epilepsy care (33). The NDDI-E-Y is particularly suited to this population as it excludes items overlapping with ASM side effects. If mild depression is identified, cognitive behavioral therapy (CBT) should be considered as first-line treatment (10); selective serotonin reuptake inhibitors (SSRIs), particularly sertraline and fluoxetine, represent first-line pharmacotherapy for moderate-to-severe cases, given their established safety profile in pediatric epilepsy and absence of seizure threshold-lowering effects (10, 34). While this discussion has focused on depression-specific screening for the reasons outlined above, a comparable symptom-tailored approach-using validated, comorbidity-specific instruments appropriate to each presenting symptom domain-should be considered for other prevalent psychiatric comorbidities identified in this cohort, such as ADHD, as part of a comprehensive psychiatric screening strategy in pediatric epilepsy care.

### 4.1 Limitations

This study has several limitations that must be considered when interpreting the results.

Its retrospective design introduces potential biases related to variability in clinical record keeping, and the lack of standardized psychiatric assessment tools (CDI-2, NDDI-E-Y) may have led to underestimation of the true prevalence of depressive and anxiety symptoms. As psychiatric symptoms were ascertained from clinical documentation rather than from systematic administration of validated screening instruments, the proportion of patients formally screened with a validated tool—or the proportion of charts containing explicit documentation of psychiatric-symptom discussion-could not be determined from the retrospective record; this affects interpretation of both the overall 30% prevalence estimate and the recommendation for systematic screening. Similarly, for the depressive/mood symptom category specifically, the retrospective record did not allow reliable differentiation between formally diagnosed depressive disorders and symptom-level presentations documented without a formal diagnosis; the reported figure (=11) therefore reflects a composite category.

The absence of longitudinal follow-up means that future prospective studies with ongoing evaluation will be needed to better characterize the onset and progression of psychiatric comorbidities, and psychosocial variables such as socioeconomic status, family support, and perceived stigma—identified in previous studies as key determinants of psychiatric risk—were not comprehensively evaluated. Similarly, seizure type and localization (e.g., temporal lobe involvement), seizure frequency, and drug-resistance status were not systematically classified as separate variables. This rules out a stratified reanalysis by seizure localization (e.g., temporal versus extratemporal, or focal versus generalized onset) for this revision; such data, prospectively collected, may reveal associations with psychiatric comorbidity not captured by the etiological G1/G2 grouping used here.

From a statistical standpoint, comparisons were limited to univariate tests (chi-squared test, Student’s *t*-test), without multivariable adjustment for potential confounders (e.g., age, sex, family history, treatment regimen) or correction for multiple comparisons across the several clinical and demographic variables assessed; both should be addressed in future analyses, as the latter increases the risk of type I error. Additionally, the 18% of patients not receiving antiseizure medication at the time of data collection were not further characterized in this dataset (e.g., by EEG findings, seizure frequency, or history of medication discontinuation after a seizure-free interval); it is possible this subgroup includes patients with a more benign or resolved course, which could not be verified retrospectively.

## 5 Conclusion

Psychiatric comorbidities, including depressive symptoms, are frequent in pediatric epilepsy and play a major role in disease burden. Depression warrants priority clinical attention given substantial clinical impact when present, atypical presentation in the pediatric age group, bidirectional relationship with epilepsy (18, 27), and significant contribution to suicide risk.

The observed association with structural and genetic abnormalities supports the hypothesis of a neurobiological basis for these comorbidities.

Early identification and systematic screening for depressive symptoms should be considered key components of epilepsy management in pediatric patients. The integration of validated screening tools [CDI-2 (29, 30) and NDDI-E-Y (31, 32)] into routine outpatient visits, alongside a multidisciplinary approach involving neurologists, psychiatrists, psychologists, and the family unit, is essential to optimize patient outcomes. Future prospective studies with longitudinal follow-up and standardized psychiatric assessment are warranted to further elucidate the genetic and neurobiological determinants of psychiatric vulnerability in pediatric epilepsy.

## Data Availability

The original contributions presented in the study are included in the article/supplementary material, further inquiries can be directed to the corresponding author.
